# Lyophilized, thermostable Spike or RBD immunogenic liposomes induce protective immunity against SARS-CoV-2 in mice

**DOI:** 10.1126/sciadv.abj1476

**Published:** 2021-12-01

**Authors:** Moustafa T. Mabrouk, Kevin Chiem, Edurne Rujas, Wei-Chiao Huang, Dushyant Jahagirdar, Breandan Quinn, Meera Surendran Nair, Ruth H. Nissly, Victoria S. Cavener, Nina R. Boyle, Ty A. Sornberger, Suresh V. Kuchipudi, Joaquin Ortega, Jean-Philippe Julien, Luis Martinez-Sobrido, Jonathan Lovell

**Affiliations:** 1 Department of Biomedical Engineering, University at Buffalo, State University of New York, Buffalo, NY 14260, USA.; 2 Texas Biomedical Research Institute, San Antonio, TX 78227, USA.; 3 Program in Molecular Medicine, The Hospital for Sick Children Research Institute, Toronto, ON, Canada.; 4 Biofisika Institute (CSIC, UPV/EHU) and Department of Biochemistry and Molecular Biology, University of the Basque Country (UPV/EHU), Bilbao, Spain.; 5 Department of Anatomy and Cell Biology, McGill University, Montreal, Quebec H3A 0C7, Canada.; 6 Animal Diagnostic Laboratory, Department of Veterinary and Biomedical Sciences, Pennsylvania State University, University Park, PA 16802, USA.; 7 Departments of Biochemistry and Immunology, University of Toronto, Toronto, ON M5S 1A8, Canada.

## Abstract

The COVID-19 pandemic has spurred interest in potent and thermostable SARS-CoV-2 vaccines. Here, we assess low-dose immunization with lyophilized nanoparticles decorated with recombinant SARS-CoV-2 antigens. The SARS-CoV-2 Spike glycoprotein or its receptor-binding domain (RBD; mouse vaccine dose, 0.1 μg) was displayed on liposomes incorporating a particle-inducing lipid, cobalt porphyrin-phospholipid (dose, 0.4 μg), along with monophosphoryl lipid A (dose, 0.16 μg) and QS-21 (dose, 0.16 μg). Following optimization of lyophilization conditions, Spike or RBD-decorated liposomes were effectively reconstituted and maintained conformational capacity for binding human angiotensin-converting enzyme 2 (hACE2) for at least a week when stored at 60°C in lyophilized but not liquid format. Prime-boost intramuscular vaccination of hACE2-transgenic mice with the reconstituted vaccine formulations induced effective antibody responses that inhibited RBD binding to hACE2 and neutralized pseudotyped and live SARS-CoV-2. Two days following viral challenge, immunized transgenic mice cleared the virus and were fully protected from lethal disease.

## Teaser

SARS-CoV-2 antigens that are displayed on liposomes and lyophilized are thermostable and protect mice from lethal virus challenge.

## INTRODUCTION

Severe acute respiratory syndrome coronavirus 2 (SARS-CoV-2) has caused a worldwide viral pandemic leading to global efforts to produce and distribute effective vaccines that prevent coronavirus virus disease 2019 (COVID-19) (*1*). The Spike protein and, more specifically, its receptor-binding domain (RBD) on the virus surface are responsible for binding to human angiotensin-converting enzyme 2 (hACE2) on the host cell. Hence, because of their central role in viral entry, the Spike and the RBD are established immunogens in SARS-CoV-2 vaccines (*2*, *3*). mRNA-based and viral-vectored vaccines encoding the Spike protein have gained regulatory approvals and are being massively deployed presently. Despite their excellent protective efficacy against SARS-CoV-2, mRNA vaccines have well-known limited thermostability, and thus, global distribution is complicated by the requirement of ultracold freezing storage temperatures (*4*). As COVID-19 vaccines are needed worldwide to immunize sufficient populations to induce global herd immunity, including in low- and middle-income countries, there is more demand for vaccine doses than at any other time in history (*5*). A potent vaccine that is effective with a lower amount of antigen per dose would increase manufacturing and distribution capacity, facilitating the supply of global needs during the pandemic.

Protein subunit vaccines are generally safe and can provide scalable production during pandemics (*6*). Nanoparticle protein-based vaccines have gained substantial attention for the Spike and RBD immunogens (*7*–*12*). Liposomes offer a safe and biocompatible vehicle for adjuvanting and delivering antigens for immunization, especially when coupled with immunostimulatory lipids such as monophosphoryl lipid A (MPLA) (*13*). Our group has previously developed a liposome-based platform that binds polyhistidine (his)–tagged proteins following simple incubation of the protein with the liposomes in aqueous conditions. The immobilization of cobalt ions within the lipid bilayer using cobalt porphyrin-phospholipid (CoPoP) enables the formation of intra-bilayer coordinate bonds with the histidine imidazole rings (*14*). This approach allows for the stable surface display of proteins with simple admixing without the need for protein engineering or chemical modifications. This strategy not only increases immunogenicity for many antigens but also displays the protein in a conformationally intact array, allowing the elicitation of high-quality functional antibody responses even when antigen and adjuvant doses are titrated down to single-digit nanograms in mouse studies (*15*, *16*). Presentation of antigens, including the RBD, on CoPoP liposomes improves uptake in antigen-presenting cells in the draining lymph node (*15*, *17*). To increase the immunogenicity of the liposome formulation, the immunostimulatory adjuvants QS-21, a saponin, and PHAD, a synthetic MPLA, are incorporated. The inclusion of PHAD has been shown to increase the functional immune response of CoPoP liposomes displaying influenza virus surface proteins (*18*), and inclusion of QS-21 in CoPoP liposomes has been shown to enhance cellular immunity (*19*). We previously reported that the his-tagged RBD was able to form a stable interaction with CoPoP liposomes that, when administered into mice and rabbits, produced antigen-specific cellular responses and antibodies that potently inhibited SARS-CoV-2 replication in mammalian cells (*17*). Inclusion of QS-21 in the bilayer of the liposomes enhanced the RBD antibody response with rabbit immunization. For these reasons, in this study, we focused on liposomes that contain CoPoP, PHAD, and QS-21 (CPQ). These liposomes are similar to the human vaccine adjuvant AS01, with the major difference being that CPQ also stably displays the antigens on the liposome surface, owing to the presence of CoPoP. While the mechanisms of the QS-21 and MPLA components of AS01 are still under investigation (*20*), they have been shown to elicit caspase-1–dependent interleukin-1β (IL-1β) and IL-18 release in antigen-presenting cells such as macrophages and dendritic cells (*21*). MPLA can stimulate Toll-like receptor ligands (TLR4) (*22*), and QS-21 activates NLRP3 inflammasomes and can disrupt lysosomal membranes (*23*).

Lyophilization, which converts liquids into solids without exposure to elevated temperatures, offers a pathway to increase the thermostability of biologics, as macromolecular biochemicals tend to be more stable in their solid form than in their liquid form (*24*). Biologics experience two types of stress during the lyophilization process, freezing and dehydration, which can result in particle aggregation and poor reconstitution (*25*). Cryo- and lyoprotectants are used to protect particles against freezing and dehydration, respectively (*26*). Disaccharides such as sucrose can act as cryoprotectants to maintain vesicle integrity during freezing (*25*, *27*). Previous studies reported thermostable protein subunit vaccines using RBD (*8*). In this study, we produce a cryoprotected, lyophilized liposomal vaccine that displays the recombinant Spike ectodomain or the RBD of SARS-CoV-2 on the particle surface. Lyophilization-dependent vaccine thermostability and protective efficacy in a K18 hACE2 transgenic mouse model of SARS-CoV-2 infection are demonstrated.

## MATERIALS AND METHODS

### Materials

His-tagged RBD (Wuhan-Hu-1 Spike amino acids 319 to 541), expressed in human embryonic kidney (HEK) 293 cells, was acquired from RayBiotech (catalog no. 230-30162). CoPoP and PoP were produced as previously described (*14*). Alhydrogel 2% aluminum gel (alum) was obtained from Accurate Chemical and Scientific Corporation (catalog no. A1090BS). The following lipids were used for forming liposomes: 1,2-dioleoyl-sn-glycero-3-phosphocholine (DOPC; Avanti, catalog no. 850375) and cholesterol (CHOL; PhytoChol, Wilshire Technologies). The only MPLA used in this study was synthetic monophosphoryl hexa-acyl lipid A, 3-deacyl (PHAD-3D6A; Avanti, catalog no. 699 855). QS-21 was obtained from Desert King. SARS-CoV-2 USA-WA1/2020 strain (GenBank: MN985325.1) was obtained from BEI Resources (NR-52281).

### SARS-CoV-2 Spike protein expression and purification

The following reagent was produced under HHSN272201400008C and obtained through BEI Resources, National Institute of Allergy and Infectious Diseases, National Institutes of Health: Vector pCAGGS containing the SARS-related coronavirus 2, Wuhan-Hu-1 Spike glycoprotein gene (soluble, with polybasic cleavage site deletion stabilized with two proline mutations at residues 682 and 685 and with a C-terminal T4 fibritin trimerization foldon and his tag), and NR-52394. This gene, containing the prefusion SARS-CoV-2 Spike ectodomain, was transiently expressed in HEK293F cells (Thermo Fisher Scientific). Cells were first split in 200-ml culture at a density of 0.8 × 10^6^ cells/ml. Fifty micrograms of filtered DNA per 200 ml of culture was mixed with FectoPRO (Polyplus Transfection) in a 1:1 ratio for 10 min at room temperature (RT). The DNA-FectoPRO mixture was added to the cells, and the cells were incubated at 125-rpm oscillation, 37°C, 8% CO_2_, and 70% humidity in a Multitron Pro shaker (Infors HT). After 6 to 7 days, cell suspensions were harvested by centrifugation at 5000*g* for 15 min. Supernatants were filtered through a 0.22-μm Steritop filter (EMD Millipore) and passed through a HisTrap Ni-NTA (nitrilotriacetic acid) column (GE Healthcare). The protein was eluted with an increasing gradient of imidazole (up to 500 mM). Ni-NTA purification was followed by a Superose 6 10/300 GL size exclusion column (GE Healthcare) in 20 mM phosphate (pH 8.0) and 150 mM NaCl buffer.

### Liposome preparation

CoPoP/PHAD/QS-21 liposomes had a [DOPC:CHOL:MPLA:CoPoP:QS-21] mass ratio of [20:5:0.4:1:0.4]. HPQ liposomes, which have a similar formulation as CPQ but contain hydrogen instead of cobalt in the PoP, served as the control liposomes and had a [DOPC:CHOL:MPLA:PoP:QS-21] mass ratio of [20:5:0.4:1:0.4]. Liposomes were prepared as recently described (*17*) using ethanol injection and nitrogen-pressurized lipid extrusion in phosphate-buffered saline (PBS), carried out at 50°C. Ethanol was then removed by dialysis against PBS twice at 4°C. QS-21 (1 mg ml^−1^) was added to the liposomes after formation at an equal mass ratio as PHAD. The final liposome concentration was adjusted to 320 μg ml^−1^ CoPoP, and liposomes were sterile filtered and stored at 4°C. Liposome sizes and polydispersity index were determined by dynamic light scattering (DLS) with a NanoBrook 90 plus PALS instrument after 200-fold dilution in PBS.

### Cell culture

For all experiments, cells were cultured and maintained at 37°C in a humidified atmosphere containing 5% CO_2_. HEK293T cells were cultured in Dulbecco’s modified Eagle’s medium (DMEM) with 10% fetal bovine serum (FBS), 1% penicillin/streptomycin, and 10 × 10^−3^ M sodium pyruvate. HEK293T-hACE2 cells were provided by M. Farzan and were cultured in DMEM with 10% FBS and 1% penicillin/streptomycin, 10 × 10^−3^ M sodium pyruvate, and puromycin (2 μg ml^−1^). Vero E6 cells (CRL-1586) were obtained from American Type Culture Collection and maintained in DMEM with 10% FBS, 1× penicillin/streptomycin/amphotericin B (Corning, catalog no. 30-004-CI), 10 × 10^−3^ M sodium pyruvate, and 1× nonessential amino acids (Corning, catalog no. 25-025-CI).

### Lyophilization

Lyophilization was carried out as previously described (*28*). Briefly, sucrose (VWR, catalog no. 97061-428) was assessed as a cryoprotectant for the RBD or Spike protein liposomal vaccine. Various concentrations of sucrose were added to either RBD or Spike liposomal formulation. Samples were frozen to −80°C overnight, before lyophilization overnight in a Labconco 700401000 FreeZone 4.5L. The condenser temperature was kept below −50°C, and the pressure was kept below 0.02 mbar. The resulting powder was characterized by scanning electron microscopy, reconstituted with deionized water, and tested for size using DLS and cryo–transmission electron microscopy.

### Ni-NTA bead competition assay

To assess RBD or Spike protein binding stability in particle form after lyophilization and reconstitution, a Ni-NTA competition assay was carried out as recently described (*17*). Ni-NTA magnetic beads (Thermo Fisher Scientific, catalog no. 88831) were added to the liposome-incubated antigens (1:4 mass ratio of total protein:CoPoP) or free antigens in PBS. Following incubation with the beads for 30 min at RT, the supernatant and beads were separated and collected using a magnetic separator (Thermo Fisher Scientific, catalog no. 12321D). Denaturing reducing loading dye was then added to the samples (supernatant and beads) and heated at 95°C for 10 min. The samples were then subjected to SDS–polyacrylamide gel electrophoresis (SDS-PAGE) using Novex 4 to 12% bis-tris acrylamide gels (Invitrogen, catalog no. NP0321BOX).

### Slot blot assay

RBD or Spike vaccine formulations in liquid or lyophilized form were stored at elevated temperatures as indicated. Lyophilized samples were reconstituted to the same concentration as the liquid samples [CoPoP (320 μg ml^−1^), RBD or Spike (80 μg ml^−1^)]. A 48-well slot blot apparatus (Bio-Rad, catalog no. M1706545) was set up as described in the manufacturer’s instructions. Fifty microliters of each sample was slowly applied into each well and then allowed to flow through a 0.45-μm nitrocellulose membrane (GE Healthcare, catalog no. 10600096) by gravity. The membrane was removed and blocked using 5% bovine serum albumin (BSA) in PBS for 30 min at RT, followed by incubating with 1000× diluted human ACE2-Fc Tag (ACROBiosystems, catalog no. AC2-H5257) for 1 hour at RT. The membrane was washed with PBS for 5 min twice and then incubated with horseradish peroxidase (HRP) anti-human immunoglobulin G (IgG; Jackson ImmunoResearch, catalog no. 109-035-098) for 30 min at RT. After incubation, the membrane was washed for 5 min with PBS two times. The membrane was developed using HRP substrate (VisiGlo HRP Chemiluminescent, catalog no. 97064-146) and imaged using a Bio-Rad ChemiDoc Imager.

### Pseudotyped SARS-CoV-2 virus

Pseudotyped SARS-CoV-2 viruses (PsVs) were produced as previously described (*17*). Briefly, HEK293T cells were seeded at 5 × 10^5^ cells ml^−1^ in a T75 flask overnight with DMEM with 10% FBS and cultured at 37°C in a humidified atmosphere containing 5% CO_2_. When the cells were approximately 60% confluent, they were transfected with the retroviral vector pQCXIX encoding firefly luciferase, a plasmid expressing murine leukemia virus (MLV) gag and pol proteins and a plasmid expressing the Spike protein of SARS-CoV-2 at a ratio of 5:5:1. Eleven micrograms of total DNA was mixed with 44 μg of polyethylenimine at RT for 20 min, and then, the mixture was slowly added to the cells. After 6 hours of incubation at 37°C, the medium was replaced with 10 ml of complete DMEM, and the culture was incubated at 32°C. After 48-hour posttransfection, the cultured medium containing PsV was harvested and passed through a 0.45-μm pore size filter and the virus supernatant was supplemented with 10 × 10^−3^ M Hepes, aliquoted, and stored at −80°C.

### Electron microscopy

For cryo–electron microscopy, holey carbon grids (C-Flat 2/1-3Cu-T) were washed with chloroform for 2 hours before sample vitrification. Before the sample was applied, grids were treated with negative glow discharge in air at 5 mA for 15 s. Sample vitrification was performed using a Vitrobot Mark IV (Thermo Fisher Scientific). Samples were applied twice to each grid to maximize the number of liposomes going inside the holes. In the first application, a volume of 3.6 μl of the liposome sample was applied to a holey carbon grid and manually blotted using the Vitrobot blotting paper (Standard Vitrobot Filter Paper, Ø55/20 mm, Grade 595) using the side window in the Vitrobot chamber. In the second application, a volume of 3.6 μl of the same sample was applied to the same holey carbon grid, and the grid was blotted once in the Vitrobot for 3 s using a blot force +1, before plunging it into liquid ethane. The Vitrobot was set at 25°C and 100% relative humidity. Grids were loaded into a Titan Krios. Movies were recorded in a Gatan K3 direct electron detector equipped with a Quantum LS imaging filter. Images were collected using SerialEM software (*29*) as 30-frame movies using 3.3-s exposures in counting mode using a total dose per movie of 49 e^−^/A^2^ at a nominal magnification of 81,000× corresponding to a calibrated pixel size of 1.09 Å. Defocus ranged for this dataset was −1.75 to −2.25 μm. Micrographs shown in the figures were obtained by merging the 30 frames in the movies after beam-induced motion correction using the MotionCor2 algorithm. For scanning electron microscopy, lyophilized liposomal antigens samples were performed using a LEO 440i (Leica Instruments, Germany) with an accelerating voltage of 15 kV. The samples were placed on double-sided conductive tape and sputter-coated with a thin layer of gold in a Pelaron SC7620 sputter coater (Ringmer, UK) for conductivity, at a covering rate of 0.51 Å s^−1^, for 180 s, using a current of 3 to 5 mA, 1 V, and at a pressure of 2 × 10^−2^ Pa.

### Murine immunization

All animal care and sample collections were approved and performed in accordance with the Institutional Animal Care and Use Committee at University at Buffalo and Texas Biomedical Research Institute (TBRI). Immunogenicity of CPQ liposomes incorporating Spike protein that was compared with Spike-containing HPQ liposome or Spike mixed with alum was performed in CD-1 mice as previously described (*17*). Alum was diluted with PBS to a concentration of 3 mg ml^−1^ and then mixed with an equal volume of diluted antigen.

Evaluation of reconstituted lyophilized CPQ liposomal vaccines alone or incorporating Spike protein or RBD antigens was performed using K18 hACE2 transgenic mice that were obtained from the Jackson Laboratory (034860) and either used directly or maintained in a breeding colony with progeny crossed with C57BL/6 mice and heterozygous pups selected following polymerase chain reaction genotyping (*30*, *31*). On days 0 and 14, mice received intramuscular injections containing 0.1 μg of RBD, Spike protein, or no antigen combined with liposome formulation [DOPC:CHOL:MPLA:CoPoP:QS-21] at [4:2:0.4:1:0.4] ratio after lyophilization with 7.5% sucrose as cryoprotectant. Liposomal vaccines were prepared by incubating the RBD or Spike protein at a concentration of 80 μg ml^−1^ with liposomes (CoPoP equivalent concentration of 320 μg ml^−1^) for 3 hours at RT before 1:1 dilution with 15% (w/v) sucrose in PBS to yield a final concentration of sucrose of 7.5% (w/v) as cryoprotectant and lyophilized. For immunization, the vaccine was reconstituted directly before the injections to a final concentration of 2 μg ml^−1^ by adding PBS and shaking the vials until solid material was not visible by eye. Serum was collected on day 28.

### Enzyme-linked immunosorbent assay

RBD or Spike protein diluted in coating buffer [1 μg ml^−1^; 28.5 mM Na_2_CO_3_ and 71.4 mM NaHCO_3_ (pH 9.6)] was used to coat 96-well plates for 2 hours at 37°C. Wells were washed and then blocked with 2% BSA in PBS containing 0.1% Tween 20 (PBS-T) for 2 hours at 37°C. Mouse sera (serially diluted 10-fold in PBS-T containing 1% BSA) were incubated in the wells for 1 hour at 37°C and then washed with PBS-T. Goat anti-mouse IgG-HRP (GenScript, catalog no. A00160) was added. Wells were washed again with PBS-T before adding tetramethylbenzidine solution (Thermo Fisher Scientific, catalog no. J60461). Antibody titers were defined as the reciprocal serum dilution at which the absorbance at 450 nm exceeded background by greater than 0.5 absorbance units.

### Confocal microscopy

RAW 264.7 cells were cultured and kept at 37°C in a humidified atmosphere containing 5% CO_2_. At 70% confluency, cells were incubated for 16 hours with Lucifer yellow (1 mg ml^−1^; Thermo Fisher Scientific, catalog no. L1177), Dextran, Alexa Fluor 647; 10,000 MW (50 μg ml^−1^; Thermo Fisher Scientific, catalog no. D22914), and Dextran, Tetramethylrhodamine, 40,000 MW (50 μg ml^−1^; Thermo Fisher Scientific, catalog no. D1842) and then washed with PBS before incubating the cells for 4 hours with QS-21 (10 μg ml^−1^) equivalent of either CPQ liposomes (QS-21 containing liposomes) before or after lyophilization. Liposomes lacking QS-21 was used at the same total lipid concentration in the same conditions as a control. The cells were lastly washed and incubated with Hoechst 33258, Pentahydrate (bis-Benzimide) (2.5 μg ml^−1^; Thermo Fisher Scientific, catalog no. H3569) before imaging using confocal microscopy at 63× magnification power.

### IgG subclass analysis

Spike protein (1 μg ml^−1^) diluted in coating buffer [28.5 mM Na_2_CO_3_ and 71.4 mM NaHCO_3_ (pH 9.6)] was used to coat 96-well plates for 2 hours at 37°C. Wells were washed and then blocked with 2% BSA in PBS-T for 2 hours at 37°C. Mouse sera (serially diluted 10-fold in PBS-T containing 1% BSA) were incubated in the wells for 1 hour at 37°C and then washed with PBS-T. Goat anti-mouse IgG-HRP (GenScript, catalog no. A00160), goat anti-mouse IgG1-HRP (SouthernBiotech, catalog no. 1073), goat anti-mouse IgG2a-HRP (SouthernBiotech, catalog no. 1083), goat anti-mouse IgG2b-HRP (SouthernBiotech, catalog no. 1093), goat anti-mouse IgG2c-HRP (SouthernBiotech, catalog no. 1077), or goat anti-mouse IgG3-HRP (SouthernBiotech, catalog no. 1103) was added. Wells were washed again with PBS-T before the addition of tetramethylbenzidine solution (Thermo Fisher Scientific, catalog no. J60461). Antibody titers were defined as the reciprocal serum dilution at which the absorbance at 450 nm exceeded background by greater than 0.5 absorbance units.

### PsV-based neutralization assay

HEK293T-hACE2 cells were seeded into 96-well plates overnight in 100 μl of media at a density of 2 × 10^5^ cells per well. Serial dilutions of sera from immunized mice were incubated with PsV at RT for 30 min. Then, 50 μl of the sera-PsV incubated samples was added to HEK293T-hACE2 cells after removing 50 μl of cultured medium. The cells were further cultured at 37°C in a humidified atmosphere containing 5% CO_2_. After 48 hours, the medium was removed from each well, and the cells were washed with 200 μl of PBS followed by the addition of 30 μl of lysis buffer (Promega, E1500). After 10 min of incubation, the lysate was transferred into a white plate, and 100 μl of luciferase assay substrate (Promega, E151A) was added. A CentroPRO LB 962 microplate luminometer was used to measure luciferase activity.

### Spike/RBD-hACE2 inhibition assay

SARS-CoV-2 cPass Surrogate Virus Neutralization Test (sVNT) Kit (GenScript, catalog no. L00847) was used to check whether post-immune sera could block the interaction between hACE2 and HRP-RBD antigen following the manufacturer’s instructions. Briefly, sera were diluted 100× with sample dilution buffer. Positive and negative controls were included in the kit, and the control vials were diluted 10×. The diluted positive and negative controls, as well as the diluted samples, were mixed with the HRP-RBD solution at a volume ratio of 1:1 and incubated at 37°C for 30 min. A total of 100 μl of these mixtures was added to an enzyme-linked immunosorbent assay (ELISA) plate precoated with hACE2, and the plate was incubated at 37°C for 15 min. The plate was washed four times to remove unbound HRP-RBD; tetramethylbenzidine solution was added, and absorbance was measured at 450 nm using a Tecan microplate reader. The percentage of inhibition was calculated as follows: % = (1 − OD_450_ post-immune sera/OD_450_ negative control) × 100.

### Live virus neutralization test

Sera neutralization of SARS-CoV-2 host cell infection was determined with a traditional VNT using SARS-CoV-2, USA-WA1/2020 strain (*32*). The live VNT protocol was generally the same as that recently used to test protective levels of neutralizing antibodies in convalescent sera from SARS-CoV-2–infected individuals (*33*). The assay was performed in triplicate for each sample, and a series of 8 to 12 twofold serial dilutions of the serum was assessed. One hundred 50% tissue culture infective dose (TCID_50_) units of SARS-CoV-2 were added to twofold dilutions of serum and incubated for 1 hour at 37°C in 5% CO_2_. The virus and serum mixture were added to Vero E6 cells in 96-well microtiter plates and grown for 3 days, after which the host cells were assessed by visual examination for virus-induced cytopathic effects on the cell monolayer. The end point of the microneutralization assay was designated as the highest plasma dilution at which all three or two of three wells were not protected from virus infection (*34*).

### Murine virus challenge

Six- to eight-week-old female K18 hACE2 transgenic mice were acquired from the Jackson Laboratory and maintained in micro-isolator cages in the Animal Biosafety Laboratory level 3 at TBRI. Mice were provided with sterile water and chow ad libitum and were acclimated for at least 1 week upon arrival before vaccination and challenge experiments. Mice were anesthetized and vaccinated intramuscularly with PBS or with 0.1 μg of RBD, Spike protein, or no antigen combined with CPQ liposomes and lyophilized and reconstituted on days 0 and 14. At 28 days after boost vaccination, the mice (*n* = 5) were challenged intranasally with 10^5^ plaque forming units (PFU) of SARS-CoV-2, USA-WA1/2020 strain and monitored daily for morbidity (body weight) and mortality (survival). Mice that have lost more than 25% of their initial body weight were considered reaching their experimental end point and were humanely euthanized. Concurrently, mice (*n* = 3) were infected and euthanized on day 2 after challenge to evaluate viral load in the nasal turbinates and lungs. Organs were homogenized in 1 ml of PBS using a Precellys tissue homogenizer (Bertin Instruments), and tissue homogenates were centrifuged at 21,500*g* for 10 min. Supernatants were collected, and viral titers were determined by plaque assay with Vero E6 cells.

### Plaque assay

To determine the viral load in nasal turbinates and lungs of 2 days postchallenged vaccinated mice, confluent monolayers of Vero E6 cells (24-well plates, 2 × 10^5^ cells per well, duplicates) were infected with 10-fold serial dilutions of supernatants from homogenates. After viral adsorption at 37°C for 1 hour, the cells were washed with PBS, then overlaid with agar, and incubated at 37°C with 5% CO_2_. Three days after infection, cells were fixed overnight in 4% paraformaldehyde and permeabilized with 0.5% Triton X-100 PBS for 10 min at RT. Plaques were detected via immunostaining using an anti–SARS 2 NP MAb 1C7 and developed with a VECTASTAIN ABC kit and a DAB HRP substrate kit (Vector laboratories) based on the manufacturer’s instructions.

## RESULTS AND DISCUSSION

We previously reported that his-tagged RBD was able to bind stably to CoPoP containing liposomes upon liquid admixing, which rendered it an effective immunogen in mice and rabbits (*17*). To expand this finding, we examined whether a recombinant Spike, the antigen used in most COVID-19 vaccines with regulatory approval, could also bind liposomes containing CoPoP, PHAD-3D6A, and QS-21 (CPQ). Figure S1A shows that the Spike protein is efficiently bound to CPQ at a mass ratio of 4:1 (CoPoP:Spike protein) without causing aggregation that would be reflected in an increase in particle size. Intramuscular immunization of mice with the Spike protein bound to CPQ elicited high Spike-specific IgG antibody titers (fig. S1B) and pseudotyped virus neutralization (fig. S1C). Significantly higher antibody titers were detected in mice immunized with Spike admixed with CPQ compared to admixed either with the identical liposomes lacking cobalt or with alum. On the basis of a Ni-NTA magnetic bead assay, the 4:1 mass ratio of CoPoP:Spike resulted in full conversion of Spike from soluble form to liposome-bound form, whereas negligible binding was observed for identical liposomes lacking cobalt, and incomplete conversion was observed with a lower mass ratio of CoPoP-to-Spike (fig. S2).

**Fig. 1.**
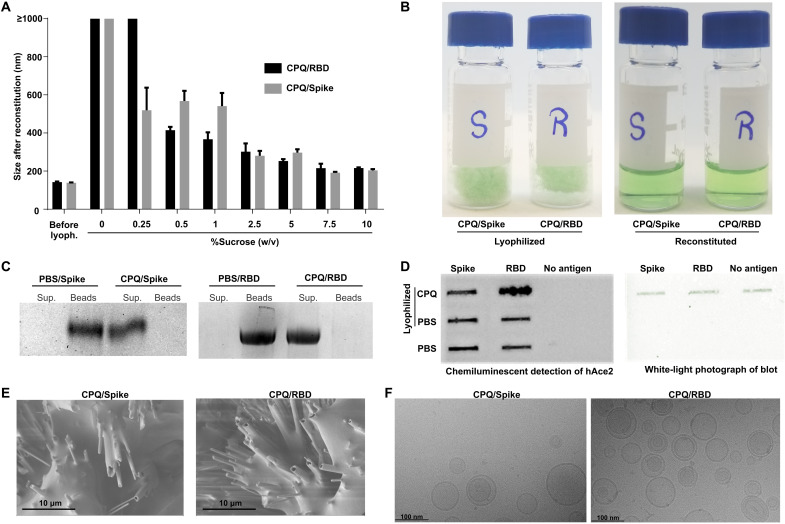
Lyophilization and reconstitution of immunogenic Spike or RBD liposomes. (**A**) Size of lyophilized CPQ formulation with varying percentages of sucrose as a cryoprotectant as measured by DLS. (**B**) Photographs of CPQ/Spike or CPQ/RBD (lyophilized with 7.5% sucrose) before and after reconstitution with water. (**C**) A Ni-NTA magnetic bead assay was used to test reconstituted CPQ/Spike or CPQ/RBD where the beads were incubated with either free protein in PBS or the reconstituted vaccine. Liposome-bound proteins remain in the supernatant (“Sup.”), but free proteins are captured by the beads. (**D**) Slot blot of soluble free antigens or lyophilized and reconstituted liposome-bound antigens demonstrating reactivity with hACE2 in the chemiluminescence blot (left) with corresponding white light photograph showing the distinct green color of CoPoP after adsorption to the membrane. (**E**) Scanning electron microscopy images of lyophilized vaccines. (**F**) Cryo–transmission electron microscopy images of reconstituted vaccines.

Next, with the intention of improving vaccine storage stability, both Spike- and RBD-bound liposomes were lyophilized. Varying amounts of sucrose were included, as this has recently been shown to be an effective cryoprotectant for CoPoP liposomes (*28*). Both antigens could effectively be displayed on liposome surfaces with simple admixture (fig. S3). As shown in Fig. 1A, without sucrose inclusion, reconstitution was inefficient, with large aggregates forming. The reconstituted powder showed a sucrose-dependent enhancement of the redispersibility index (RI; values close to unity reflect efficacy of reconstitution), reaching 1.09 ± 0.02 and 1.07 ± 0.01 for liposome-displayed RBD and Spike, respectively, when 7.5% sucrose was used. Greater concentrations of sucrose did not lead to further enhancement of the RI, so 7.5% was selected for all subsequent lyophilization procedures.

The lyophilized powder formed a porous cake at the bottom of the glass vial, as shown in (Fig. 1B). Within minutes of water addition, a uniform green translucent liquid was observed. Lyophilization and reconstitution did not adversely affect antigen biding to the liposomes, as incubation of the reconstituted vaccines with Ni-NTA magnetic beads did not reveal the presence of any nonbound protein in a Ni-NTA challenge assay (Fig. 1C). Conformational integrity of the proteins bound to the liposomes after lyophilization was assessed by slot blot. Reconstituted vaccines were adsorbed on the surface of a nitrocellulose membrane and then probed for reactivity with hACE2. As shown in Fig. 1D, both Spike- and RBD-bound liposomes were recognized by the SARS-CoV-2 human receptor hACE2.

Further analysis of the lyophilized cake was done by imaging the powder using a scanning electron microscope, which revealed a generally amorphous and porous nature as shown in Fig. 1E. Channels were apparent in the cake, which may potentially reflect the presence of frozen water channels that sublimated during the freeze-drying process, contributing to the rapid dissolution as the water goes back to these pores during reconstitution (*35*). After reconstitution, morphological integrity of the liposomes was observed using cryo–transmission electron microscopy and showed that the liposomes were spherical with no evidence of fusion or coalescence due to lyophilization (Fig. 1F). One functional method that QS-21 exerts its mechanism of action is thought to relate to lysosomal disruption (*23*). To assess whether this property of QS-21 was affected by lyophilization, lysosome disruption induced by CPQ before and after lyophilization was compared to the same formulation lacking QS-21 (CP). Fluorescence micrographs of murine macrophages showed that that CPQ, but not CP, effectively induced lysosomal cargo release into the cytoplasm, regardless of whether the liposomes had been lyophilized or not (fig. S4).

**Fig. 2.**
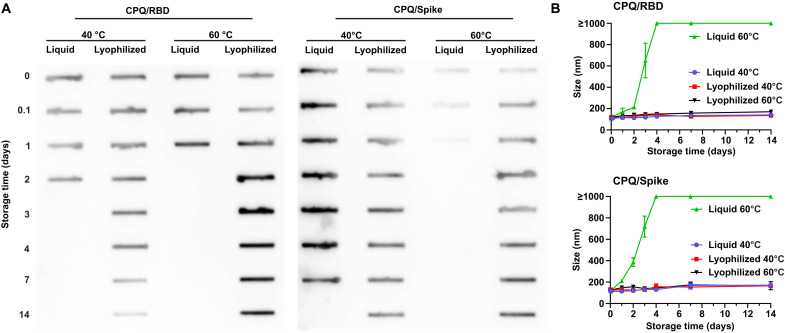
Thermostability of lyophilized RBD and Spike liposomal vaccines. CPQ was incubated with Spike or RBD to form particles, lyophilized, and then assessed following storage at elevated temperatures. (**A**) Slot blot detection of vaccine reactivity with hACE2 (representative image shown from *n* = 3 separate experiments). (**B**) Size stability of the stored liposomes in various conditions (mean ± SD for *n* = 3 samples). All measurements for lyophilized samples were recorded following vaccine reconstitution.

To assess the accelerated thermostability of the vaccines, liquid and lyophilized Spike or RBD liposomes were incubated at 40° or 60°C for up to 14 days. After incubation, the ability of the immunogens to recognize ACE2 was assessed by slot blot (Fig. 2A). When incubated at 40°C for up to 14 days, the lyophilized formulation conserved the conformation of the proteins, which recognized ACE2 upon reconstitution. This high stability was also observed at 60°C. In the case of the RBD, the protein was functional for 7 days when incubated at 60°C while the Spike protein was stable for the entire duration of the experiment. In contrast, the integrity of the liquid formulations was compromised at day 3 (RBD) or day 7 (Spike) at 40°C. On the other hand, at 60°C, the liquid formulation based on the RBD and Spike protein failed at day 2 and in less than 1 day, respectively.

Particle size stability was also assessed during storage at elevated temperatures. Only the lyophilized formulations maintained colloidal stability (upon reconstitution) during the incubation period at 60°C. In the case of liquid formulations, aggregation was detected as early as on day 2 (Fig. 2B). The binding stability of the protein to the liposomes remained the same upon incubation at high temperature for 14 days with no signs of proteolysis in the lyophilized form (fig. S3). On the other hand, the proteins in the liquid formulation were not detected on the SDS-PAGE after 14 days, likely because of protein precipitation due to exposure of the hydrophobic core during prolonged heating (*36*). Although further long-term stability studies should be performed, these data suggest that the lyophilized formulations would not require extremely low temperatures for storage, which may facilitate stockpiling and transportation.

**Fig. 3.**
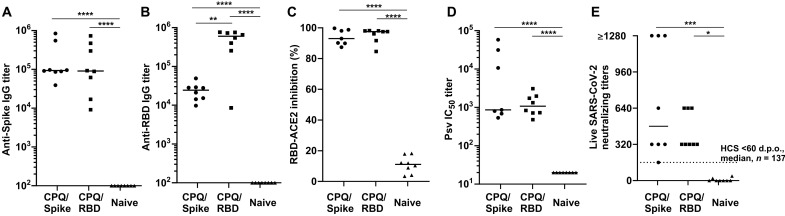
Lyophilized CPQ/Spike or CPQ/RBD vaccines induce functional antibodies in K18 hACE2 transgenic mice. K18 hACE2 transgenic mice were immunized intramuscularly on days 0 and 14 with reconstituted vaccines (0.1 μg of antigen), and serum was assessed on day 28. Anti-Spike (**A**) or anti-RBD (**B**) IgG titer measured by ELISA. (**C**) Inhibition of RBD interaction with hACE2 by the indicated diluted post-immune sera was measured by surrogate virus neutralization assay. (**D**) Pseudotyped SARS-CoV-2 and (**E**) live SARS-CoV-2 virus neutralization titers of post-immune sera. IC_50_, median inhibitory concentration. The dashed line in (E) shows SARS-CoV-2 neutralization median in the same assay using convalescent sera (HCS) from 137 SARS-CoV-2–infected humans, <60 days post onset (d.p.o.) of symptoms (*33*). Data were analyzed by one-way analysis of variance (ANOVA) followed by Tukey’s test. *P < 0.05, **P < 0.01, ***P < 0.005, and ****P < 0.001.

The lyophilized vaccines were used to immunize K18 hACE2 transgenic mice (*37*) following a prime-boost regimen with 14 days between the two intramuscular doses of 0.1 μg of antigen (containing 0.4 μg of CoPoP, 0.16 μg of PHAD, and 0.16 μg of QS-21). Vaccines were reconstituted immediately before immunization. As shown in Fig. 3 (A and B), both vaccines induced high serum antibody titers against the Spike and RBD after the boost immunization. The functional activity of the elicited antibodies was first tested by monitoring their ability to block RBD-ACE2 binding in a surrogate virus neutralization assay. Incubation of the CPQ Spike or RBD postvaccination sera inhibited RBD-ACE2 interaction, suggesting the induction of functional antibodies (Fig. 3C). The postvaccination sera were confirmed to effectively neutralize a pseudotyped virus expressing the Spike protein and live SARS-CoV-2 (Fig. 3, D and E). In immunization studies done using lyophilized Spike protein or RBD CPQ liposomes, IgG isotyping revealed a balanced T helper cell 1 (T_H_1)/T_H_2 response, as shown in figs. S5 and S6. In the current study, both liposome-displayed antigens (the RBD and Spike) potently induced neutralizing antibodies with two injections of 0.1 μg of antigen. This is in contrast to a prior study of the RBD and Spike adjuvanted with AddaVax, with three injections of 5 to 50 μg of antigen, in which the Spike demonstrated superior compared to the RBD (*38*). The higher immunogenicity of particle-based approach may relate to the putative hapten-like behavior of the small and compact RBD, which we previously demonstrated exhibits enhanced uptake into antigen-presenting cells in particle format (*17*).

**Fig. 4.**
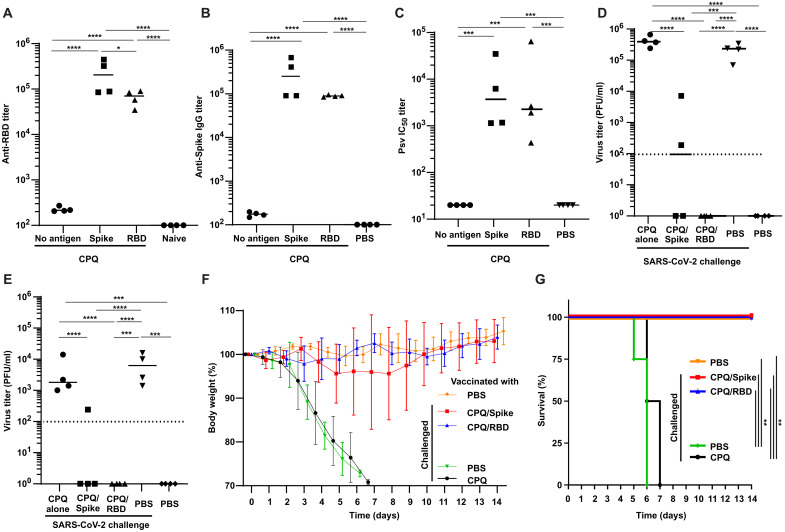
Lyophilized CPQ/Spike or CPQ/RBD liposomes protect K18 hACE2 transgenic mice against lethal SARS-CoV-2 challenge. Anti-Spike (**A**) or anti-RBD (**B**) IgG titer at day 28 after intramuscular vaccination with reconstituted lyophilized vaccine (0.1 μg of antigen) on days 0 and 14. (**C**) Pseudotyped SARS-CoV-2 virus neutralization assay of the prechallenge, post-immune sera. Viral load, as assessed by plaque assay from the nasal turbinate (**D**) and the lung tissue (**E**) at day 2 after SARS-CoV-2 challenge. (**F**) Percent body weight change calculated from days 0 to 14 after challenge. (**G**) Survival curve. For (A to E), log_10_-transformed titer was analyzed by one-way ANOVA test, followed by Tukey’s comparisons. For (G), data were analyzed by log-rank test. *P < 0.05, **P < 0.01, ***P < 0.005, and ****P < 0.001.

Encouraged by the strong antibody responses induced by the reconstituted vaccines, another study was conducted in which immunized K18 hACE2 transgenic mice were intranasally challenged with a lethal dose (10^5^ PFU) of SARS-CoV-2. Using the same 0.1-μg antigen dosing and immunization schedule, prechallenged, vaccinated mice again exhibited high IgG against the RBD (Fig. 4A) and Spike (Fig. 4B) that neutralized pseudotyped virus (Fig. 4C). Mice were then challenged with SARS-CoV-2 on day 28. Two days following the challenge, one cohort was euthanized, and viral loads in the nasal turbinate and lungs were assessed. Virus levels were significantly lower in the CPQ/Spike and CPQ/RBD immunized mice than control mice immunized with PBS or with CPQ lacking antigen (Fig. 4, D and E). Most immunized mice had undetectable levels of virus. The suppression of viral loads in the vaccinated mice was further corroborated with 100% survival of the vaccinated mice with no significant weight loss (Fig. 4, F and G).

While this study established that a liposome-displayed Spike or RBD is amendable to simple lyophilization, which confers thermostability, and induces full protection in the transgenic hACE2 mouse model with only 0.1 μg of antigen, limitations should be noted. Only the Wuhan-Hu-1 Spike protein and RBD antigen sequences were used, and the efficacy against emerging variants of concern, which are of high importance, was not assessed. Furthermore, factors relevant to COVID-19 immunity, including sex, age, and comorbidities such as cardiovascular disease or diabetes, were not evaluated at this time. This could be addressed in the future by using mouse-adapted SARS-CoV-2 and appropriate mouse animal models. Furthermore, the thermostability was assessed only for relatively short periods without controlled humidity. Although lyophilization may simplify some elements of vaccine production and distribution, it may also complicate actual vial production, considering that lyophilization requires batch processing. This study did not assess the safety of the vaccine, although we previously found that the vaccine was well-tolerated in a mouse toxicity study (*17*). The inclusion of cobalt in CoPoP should also be noted. Another cobalt-chelated porphyrin-like macrocycle, cobalamin (vitamin B12), has a strong safety record in humans and has been assessed in human testing as an antidote to cyanide poisoning at intravenous doses of 5 g (*39*). In comparison, the mouse CoPoP dose in this study was 0.4 μg, which is several orders of magnitude smaller on a milligram per kilogram basis and corresponds to a chelated cobalt dose of approximately 0.02 μg. We previously found that immunization with CoPoP liposomes did not increase serum cobalt levels detectably (*17*). At present, there are no immediate plans to further develop a lyophilized SARS-CoV-2 vaccine with CoPoP. However, a CoPoP and MPLA liposome-based RBD vaccine has recently entered phase 1/2 clinical trials in South Korea (ClinicalTrials.gov identifier: NCT04783311) with the trade name EuCorVac-19. Thus, the studies presented here underscore the future long-term potential of this particle-based vaccine adjuvant system.

CoPoP liposomes decorated with Spike or RBD were demonstrated to be amenable to lyophilization with use of sucrose as a cryoprotectant. This resulted in enhanced thermal stability while maintaining high immunogenic potency in mice. Further work is required to assess the longer-term stability of this system. In general, thermostability is desirable as it facilitates worldwide distribution and storage with reduced cold chain restrictions, one of the challenges facing current COVID-19 vaccines. Lyophilization of these SARS-CoV-2 antigen particles is compatible with simple reconstitution in sterilized water before injection. Besides being thermostable, a potent vaccine with a low antigen dosage regimen can increase manufacturing output, expediting production capacity to supply global need during a pandemic to developing countries that face challenges adhering to strict cold chains required for vaccines stored in frozen or ultralow-temperature frozen conditions.

## Notes

View/request a protocol for this paper from *Bio-protocol*.
