# Supplementary material for: Lyophilized, thermostable Spike or RBD immunogenic liposomes induce protective immunity against SARS-CoV-2 in mice
Source: Sci Adv. 2021 Dec 1;7(49):eabj1476. doi: 10.1126/sciadv.abj1476 (PMC8635435; doi:10.1126/sciadv.abj1476)
Supplement: Supplementary file 1 — Figs. S1 to S6 [file sciadv.abj1476_sm.pdf]

Science Advances
AAAS

Supplementary Materials for

Lyophilized, thermostable Spike or RBD immunogenic liposomes induce protective immunity against SARS-CoV-2 in mice

Moustafa T. Mabrouk, Kevin Chiem, Edurne Rujas, Wei-Chiao Huang, Dushyant Jahagirdar, Breandan Quinn, Meera Surendran Nair, Ruth H. Nissly, Victoria S. Cavener, Nina R. Boyle, Ty A. Sornberger, Suresh V. Kuchipudi, Joaquin Ortega, Jean-Philippe Julien, Luis Martinez-Sobrido, Jonathan Lovell*

*Corresponding author. Email: jflovell@buffalo.edu

Published 1 December 2021, *Sci. Adv.* **7**, eabj1476 (2021)
DOI: 10.1126/sciadv.abj1476

This PDF file includes:

Figs. S1 to S6

Supporting Information:

Lyophilized, Thermostable Spike or RBD Immunogenic Liposomes Induce Protective Immunity Against SARS-CoV-2 in Mice

Moustafa T. Mabrouk, Kevin Chiem, Edurne Rujas, Wei-Chiao Huang, Dushyant Jahagirdar, Breandan Quinn, Meera Surendran Nair, Ruth H Nissly, Victoria S. Cavener, Nina R. Boyle, Ty A. Sornberger, Suresh V. Kuchipudi, Joaquin Ortega, Jean-Philippe Julien, Luis Martinez-Sobrido, Jonathan Lovell

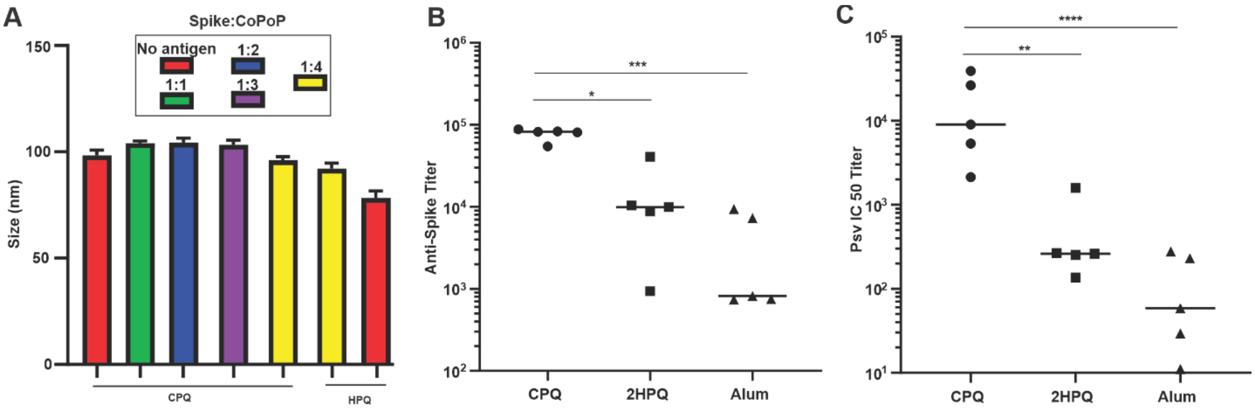

Fig. S1: Particle formation and immunogenicity of his-tagged Spike protein A) Size of liposomes CPQ (CoPoP/PHAD/QS-21) or HPQ liposomes (PoP/PHAD/QS-21) following incubation with Spike protein. All experiments were done after 3 hr incubation of the spike protein with the liposomes **B, C)** Day 28 serum antibody characterization from mice vaccinated using 100 ng equivalent of Spike protein at 1:4 mass ratio of Protein: CoPoP at day 0 and day 14. **B)** Anti- Spike protein titer as measured by ELISA. **C)** Pseudotyped SARS-CoV-2 neutralization assay.

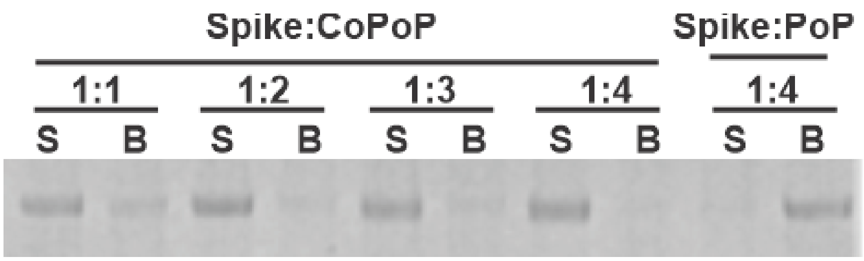

Fig. S2: Binding of the Spike protein to CoPoP liposomes. Nickel-nitrilotriacetic acid (Ni-NTA) magnetic beads challenge assay showing binding capacity and stability of Spike protein to liposomes containing cobalt (CoPoP) at various protein: CoPoP mass ratios versus liposomes lacking cobalt (PoP) at highest protein: CoPoP mass ratios. (S) stands for supernatant (liposome-bound fraction) while (B) stands for the Ni-NTA beads fraction (liposome-unbound fraction)

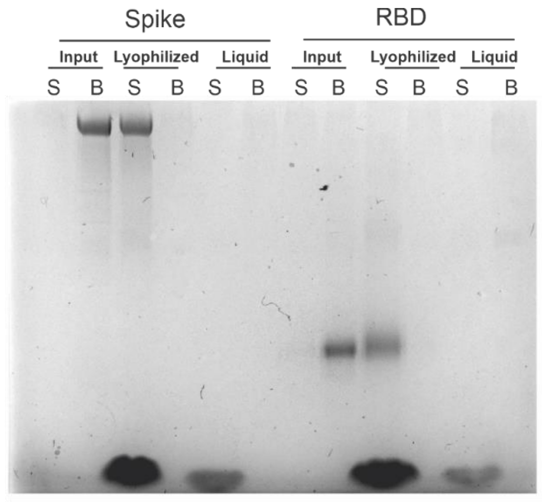

Fig. S3 Ni-NTA challenge for thermal stability. Nickel-nitrilotriacetic acid (Ni-NTA) magnetic beads challenge assay showing binding capacity and stability of RBD or Spike protein to CPQ liposomes either in liquid or lyophilized form after being incubated at 60 °C for 14 days. “S” stands for supernatant (liposome-bound fraction) while “B” stands for beads fraction (liposome-unbound fraction). “Input” represents the protein without CPQ.

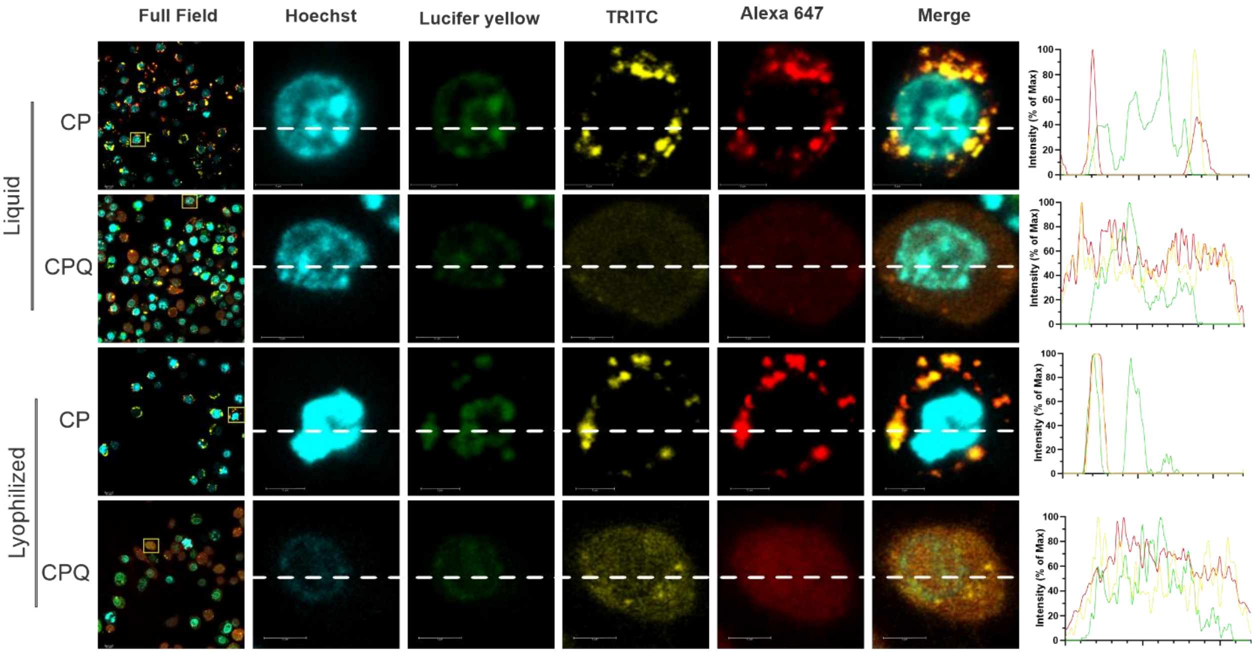

Fig. S4. QS-21-containing CoPoP liposomes promote lysosome destabilization. RAW 264.7 Cells were incubated with Lucifer Yellow (1mg/ml), 10 kDa dextran Tetramethylrhodamine isothiocyanate (TRITC) (50 µg/mL) and 40 kDa dextran-Alexa647 (50µg/ml) for 16 h then washed 6 times with PBS before stimulating with 10 µg/ml QS-21-equivelant liposomes (CPQ) as liquid before lyophilization or after Lyophilization and reconstitution for 4 h. Liposomes lacking QS-21

was used as a control at the same concentration before/after lyophilization. Representative single cells are shown at higher magnification. The localization of fluorescent markers across the dashed line was analyzed using ImageJ. Scale bar is 10 μm in larger field of view and 5 μm for the single cell images.

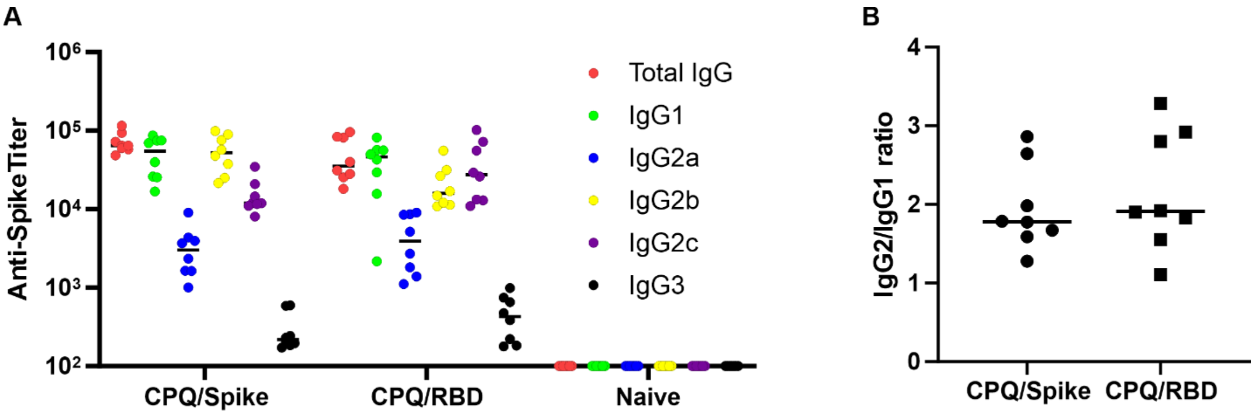

Fig. S5: IgG subclass analysis after immunizing K18 hAce2 transgenic mice with 0.1 μg lyophilized CPQ/Spike or CPQ/RBD vaccines A) IgG subtypes titers, B) IgG2/IgG1 ratios calculated as followed: titer value (IgG2a+IgG2b+IgG2c) /titer value IgG1.

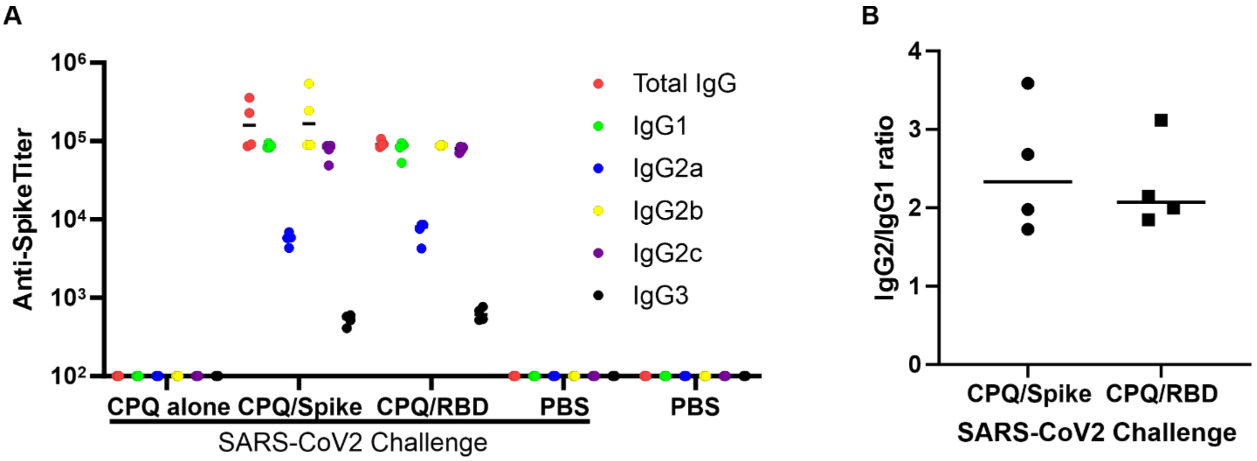

Fig. S6: IgG subclass analysis after immunizing SARS-CoV-2 challenged K18 hAce2 transgenic mice with 0.1 μg lyophilized CPQ/Spike or CPQ/RBD vaccines A) IgG subtypes Titers, B)IgG2/IgG1 ratios calculated as followed: titer value (IgG2a+IgG2b+IgG2c) /titer value IgG1.
